# Exploring the Clinical and Genetic Landscape of Angelman Syndrome: Patient-Reported Insights from an Italian Registry

**DOI:** 10.3390/jcm13123520

**Published:** 2024-06-16

**Authors:** Pier Luigi Carriero, Rosalia Zangari, Eleonora Sfreddo, Arianna Ghirardi, Arrigo Schieppati, Tiziano Barbui, Francesco Biroli

**Affiliations:** 1 FROM Research Foundation ETS, Papa Giovanni XXIII Hospital, 24127 Bergamo, Italy; plcarriero@fondazionefrom.it (P.L.C.); rzangari@fondazionefrom.it (R.Z.); esfreddo@fondazionefrom.it (E.S.); aghirardi@fondazionefrom.it (A.G.); tbarbui@fondazionefrom.it (T.B.); 2 Clinical Research Centre for Rare Diseases “Aldo and Cele Daccò”, Mario Negri Institute for Pharmacological Research, 24020 Ranica, Italy; arrigo.schieppati@marionegri.it

**Keywords:** Angelman syndrome, epilepsy, genotype, maternal 15q11–q13 region deletion, rare disease, patient-driven registry, patient registry

## Abstract

**Background:** The Angelman Syndrome Registry (RISA) was developed as a retrospective study with the following objectives: to evaluate the clinical history of individuals with Angelman Syndrome (AS) in Italy and compare it with the existing literature; to investigate the feasibility of gathering data by directly involving participants in the data collection process; and to explore the relationship between different symptoms and genotypes. **Methods:** Established in 2018, RISA enrolled a total of 82 participants, with 62 (75.6%) providing complete data. Demographic, clinical, and genetic information was collected using electronic case report forms. Descriptive statistics characterized the sample, while associations between genotype and clinical characteristics were examined. **Results:** Descriptive analysis revealed a median participant age of 8.0 years, with males comprising 48.8% of the sample. Deletion (58.1%) was the most common genotype. The majority (82.2%) experienced epilepsy, with seizures typically onset before 3 years of age. Most patients (86.2%) required multiple anti-epileptic drugs for control, with generalized tonic–clonic seizures and atypical absence seizures being most prevalent. The deletion group exhibited more severe developmental delays and a trend towards higher seizure severity. Sleep problems affected 69.4% of participants, characterized by difficulties in sleep onset and maintenance. **Conclusions:** This study offers valuable insights into the clinical history and genetic characteristics of AS in Italy, consistent with the prior literature. Additionally, it underscores the efficacy of patient registries in capturing comprehensive data on rare diseases such as AS, highlighting their potential to advance research and enhance patient care.

## 1. Introduction

Angelman syndrome (AS, OMIM 105830) is a rare genetic neurodevelopmental disorder caused by loss of function in neurons of the ubiquitin-protein ligase E3A gene (UBE3A), an imprinted, maternally expressed gene in chromosomal region 15q11–q13 [1,2,3,4,5].

The estimated prevalence of AS is 1/10,000–25,000 [6,7,8,9], although the exact prevalence is unknown. As a result of the loss of UBE3A gene function, patients with AS have moderate to severe developmental delay, intellectual disability, motor or balance impairment, absent or impaired speech, sleep disturbances, seizures, and several behavioral characteristics [9,10,11]. The life expectancy of most AS patients appears to be normal. However, they cannot live independently, and various symptoms may worsen over time. Phenotypic heterogeneity appears to be due to molecular etiologies [12,13,14,15]. Different molecular classes of defects impair the functionality of UBE3A: 60–70% of AS cases are caused by a maternal deletion of the 15q11–q13 region; 15–20% of AS cases are caused by a paternal uniparental disomy of chromosome 15, altered methylation in the imprinting center, a chromosomal translocation, and a point mutation; in 10% of cases, the genetic alterations remain unknown [1,2,3,4,5,16]. Treatments target the symptoms without addressing the underlying etiologic mechanisms by which UBE3A deficiency leads to AS [17]. Available treatments focus on controlling seizures and managing the physical and behavioral symptoms of the disease, requiring a multidisciplinary team. Therefore, it is crucial to obtain a more detailed overview of the overall epidemiology and symptomatology of AS, with a focus on epilepsy (which affects 80–90% of cases) and neurodevelopmental outcomes to target future interventions [18].

In recent years, the use of registries has gained importance in supporting clinical and epidemiological research [19].

Voluntary direct reporting by patients (patient-driven registries or patient self-reported registries) proves that data from patients/families are rich and important sources of information that can be used to address the challenges related to rare diseases [20,21,22,23,24].

These registries are tools used to collect and organize clinical and demographic data on individuals with rare diseases to support scientific research, improve understanding of the disease, and facilitate the development of new treatments and therapies. This adaptation is part of the broader trend toward research involving patient-centered outcomes, which emphasizes the inclusion of the patient perspective in clinical research [25,26].

There is a growing consensus that they provide a more comprehensive and collaborative approach to rare diseases, integrating classical physician-driven data collection with patient-reported outcomes (PROs). Together with clinician-reported data, a patient-driven registry provides a dynamic framework for improving the robustness, scope, and quality of data collection [20,27,28,29,30].

This paper describes the results of the Italian Angelman Syndrome Registry (RISA), a patient-driven registry for Italian persons affected by AS.

Studying Angelman Syndrome in Italy holds significance due to the potential influence of specific environmental and health conditions. National population studies on AS were not available at the time the registry was launched, emphasizing the need for independent initiatives supported by patient associations. This study seeks to corroborate previous findings by actively involving the families of individuals with AS. It aims to fill a gap in Italy, where existing studies are often based on limited case series from referral centers. By including a sample of AS patients from across Italy, this study aims to provide additional insights into the Italian context that can complement research efforts and contribute to a more comprehensive understanding of the syndrome.

The research question was descriptive in nature, with the main objectives being: (1) to evaluate the clinical history of individuals with Angelman Syndrome in Italy and to compare it with the existing literature, (2) to investigate the feasibility and effectiveness of gathering data while directly involving participants in the data collection process, and (3) to investigate the association between different symptoms, such as epilepsy and sleep disorders, with specific genotypes.

## 2. Materials and Methods

### 2.1. Study Design

This study utilized data from the Italian Angelman Syndrome Registry (RISA), a patient registry designed to collect comprehensive clinical and genetic information about individuals diagnosed with Angelman syndrome (AS) in Italy. The registry was designed as a retrospective study with the direct involvement of participants (parents/guardians) in data collection (patient-driven methodology). The registry was established in February 2018 and was accessible online until February 2021 (a 3-year activity period, see Supplementary S1 for more details).

### 2.2. Eligibility and Enrollment

All individuals of any age who lived on Italian territory and with a diagnosis (evidenced by genetic tests) of AS were eligible for participation. Participation was voluntary and was mediated through the involvement of AS associations. Written informed consent was obtained for participation. As the cognitive impairments of AS patients limit their participation, parents/guardians were directly involved. Restricting the study to Italian residents ensured a homogeneous sample for analysis and addressed regulatory concerns. The rationale for the exclusion criteria was to maintain relevance to AS by ensuring that all observations were pertinent to the condition.

### 2.3. Regulatory and Ethical Concerns

The Registry complied with Directive 95/46/EC (General Data Protection Regulation) on data protection and received appropriate ethical approval from the Internal Review Board (9 February 2018). Information on the Registry can be found at https://clinicaltrials.gov/study/NCT03650569 (ID: NCT03650569 accessed on 14 June 2024) and on the Ophanet website (https://www.orpha.net/it/research-trials/registry/563439?name=&mode=&country= (accessed on 14 June 2024)). This paper follows the standards of data reporting for retrospective studies [31].

### 2.4. Data Source

Data collection was centralized and performed through a dedicated Electronic Data Capture (EDC) system capable of supporting electronic case report forms (e-CRFs), which were set up for this purpose. Data were entered into electronic forms directly by the participants through their mobile devices or computers. The forms contained in the eCRF were designed to cover 6 key areas (Figure S1). Demographic data were collected separately. Information on medical history, diagnosis and genetic results, behavior and development, sleep and epilepsy, and general aspects (strabismus/other vision problems, gastrointestinal problems, skeletal/muscles disorders, respiratory problems) was recorded.

Information was primarily reported by the parents or caregivers of individuals with AS, including data from medical professionals such as geneticists, neurologists, and pediatricians to increase accuracy and completeness.

Common items identified in the scientific literature were included. The following variables were listed to capture the history of the diagnosis: abnormal neurological examination (during clinical assessments of neurological functions, electroencephalograms, or imaging studies), ataxia, developmental delay (especially motor development), lack of speech, microcephaly, seizures, unusual behavior, and jerky limb movements.

The variables used to record the genetic results were: (1) type of genetic tests, i.e., DNA methylation test; chromosomal microarray (CMA), mutation; FISH; DNA sequencing and (2) the data and results for each test.

Epilepsy was captured by listing all the seizure types according to the International League Against Epilepsy (ILAE) 2017 classification [32].

A self-reported form (patient-reported outcomes, PROs) [30,33] was used for sleep disturbances, based on the following items: quantity (duration), quality (problems or disorders), and regularity (bedtime schedule).

Reliability was ensured by reviewing the source documents and providing feedback to participating users (e-queries resolutions). To enhance the accuracy of the self-reported data and mitigate potential biases, several measures were implemented, see Supplementary S1.

### 2.5. Data Analysis

Continuous variables were expressed as median and interquartile range (IQR). As no variables exhibited a normal distribution (using Shapiro–Wilk tests), non-parametric tests were used. Categorical variables were expressed as absolute numbers and percentages, with data categorized as Yes (Y), No (N), Not Applicable (NA), Unknown (UNK). With the exception of demographic data and PROs, only completed user data were analyzed. The final dataset (Table S1) was compared with the Global Angelman Syndrome Registry (Global Registry for short) [34], given that the two studies had similar methodologies. The comparison was limited to variables common to both registries (cutoff: 3-year activity of the registry or 1-year activity of the Global Registry). Differences between registries were assessed using the chi-square test or Fisher’s test (if applicable). The group of non-deletions included: uniparental disomy, imprinting defects, and mutation and non-deletion cases. The Mann–Whitney U test, chi-square test, or Fisher’s test (if applicable) were used to compare the characteristics of AS patients. Statistics were performed using STATA software, version 16 (StataCorp LP, College Station, TX, USA).

## 3. Results

From 15 February 2018 to 15 February 2021, 82 participants (parents/guardians of individuals with AS, subjects enrolled in the study) completed the registration process and were included. The sample consisted of 62 (75.6%) individuals with completed data entry and submitted source documents (Table 1). Available data from the original 82 participants, including those who did not complete data entry, were used for sample description (Table 1).

### 3.1. Demographic and Clinical Characteristics

The median age was 8.0 (IQR: 3.0–17.7, range 0–54.0), the male-to-female ratio was 1:1.05, and 98.8% were of Caucasian origin. The age groups are listed in Table S1. The high proportion of younger participants (particularly in the age group of 3–5, 23.2%) and the male-to-female ratio were consistent with available data from the Global Registry (Table S1). The participants came from 17 Italian regions.

Among the behavioral and developmental characteristics listed (see the Data Source paragraph), a delay in movement development (57/62, 92.0%) and speech disorders (49/62, 79.0%) were the first causes leading to diagnosis and were also reported as the main acquired delays (Table 1). Genetic counseling was considered by 62.9% (39/62) of participants to confirm the diagnosis. On average, genetic diagnoses were made after 2 years.

The common reported reasons for hospitalization (Table 1) due to comorbidities were tonsillectomy and adenoidectomy, dental procedures, orthopedic procedures, conditions that may require corrective surgical interventions, epilepsy and seizures, and bronchiolitis.

According to the Global Registry, a 15q11–q13 deletion was the most frequent genotype, followed by uniparental paternal disomy and mutation. The genetic diagnoses (Table S1) were deletion in 58.1%, with the remaining showing paternal uniparental disomy and mutation (~34.0%), an imprinting defect, or abnormal DNA methylation/undefined cases (~8.0%). The distribution of genotype classes was also comparable to more recent results [35] and data collected by previous studies [18,36].

### 3.2. Clinical Characteristics and Genotype Association

The most frequently reported symptoms by parents were epilepsy and sleep problems (Table 2).

Epilepsy was described in 82.2% of participants, with the onset typically occurring before 3 years of age, despite 13 patients still below the median age of onset at the time of registration. Three subjects had their first seizure in the first year of life, and 25 developed symptoms within the second year and a half. The majority of patients in this sample had generalized epilepsy (82.3%), and 12.0% also had partial epilepsy. Epilepsy aggravated by fever occurred in 39.2%. Overall, 44.0% of patients had more than one seizure type (mean, 1.6 types), with a prevalence of generalized tonic–clonic seizures and atypical absences. In fifteen subjects, the first seizure occurred during a febrile convulsion. Among those with seizures, 86.2% required multiple anti-epileptic drugs (AEDs) for control (for example, drugs targeting GABA-ergic signaling, such as valproic acid, clonazepam, benzodiazepines, used either as a monotherapy or in combination). Severe epilepsy with drug-resistant seizures was described in four cases. Analysis of the onset and severity of epilepsy (Table 2) showed that: (1) age at onset of epilepsy (2 years) was associated with the deletion subtype; (2) the deletion subtype showed a trend toward the highest rates of severe epilepsy and multiple seizures. These results were in line with the literature [11,35,36,37,38].

**Table 2:** Characteristics of AS patients (deletion vs. non-deletion).

	Total (*n* = 62)	Deletion (*n* = 36)	Non-Deletion (*n* = 26) ^1^	*p*-Value
Age at diagnosis (year), median (IQR)	2.0 (1.1–3.0)	1.3 (0.9–2.0)	3.0 (2.0–6.3)	**<0.001**
Age at registration (year), median (IQR)	8.0 (3.0–18.0)	4.0 (2.3–16.0)	12.5 (3.8–20.8)	**0.031**
**Epilepsy, *n* (%) ^3^**	51 (82.2)	31 (86.1)	20 (76.9)	0.550
Age at onset of epilepsy, median (IQR)	2.5 (2.0–3.0)	2.0 (1.8–3.0)	3.0 (2.0–6.0)	**0.014**
Frequency of seizures (year), median (IQR) ^2^	1.0 (1.0–5.0)	2.0 (0.2–5.8)	1.0 (1.0–5.0)	0.898
Anti-epileptic Treatment, *n* (%)	44 (86.2)	29 (93.5)	15 (75.0)	0.143
Febrile seizures [38], *n* (%)	20 (39.2)	15 (48.4)	5 (25.0)	<0.05 *
Generalized seizures, *n* (%)	42 (82.3)	25 (80.6)	17 (85.0)	0.982
Focal seizures, *n* (%)	6 (12.0)	3	2	-
Tonic–clonic seizures, *n* (%)	22 (44.0)	19 (61.3)	3 (15.0)	<0.01 *
Absence seizures, *n* (%)	28 (56.0)	17 (54.8)	11 (55.0)	0.568
Multiple seizure types ^3^, *n* (%)	22 (44.0)	20 (64.5)	2 (10.0)	<0.01 *
**Sleep problems**				
Problems with bedtime schedule, *n* (%)	43 (69.4)	23 (63.9)	20 (76.9)	0.346
Night sleep latency (minutes), median (IQR)	30.0 (−90.0)	35.0 (5.0–105.0)	25.0 (6.3–105.0)	0.598
Night wakings, median (IQR)	2.0 (1.0–3.0)	1.0 (1.0–3.0)	2.0 (1.0–3.0)	0.478

^1^ “Deletion” and “Non-Deletion” refer to different genotypic groups. Non-Deletion group: uniparental disomy, imprinting defects, point mutation and abnormal DNA metilation or undefined cases. ^2^ Number of seizure episodes experienced within a one-year period. ^3^ tiple seizure types: At least two main types of recurrent seizures, including generalized tonic–clonic seizures, typical absences, atypical absences, focal seizures, convulsive status epilepticus, and status epilepticus during febrile seizures. Variables related to epilepsy and sleep problems are in bold. Significant *p*-Value (*p* < 0.05) are in bold. ^3^ The table compares the frequencies of positive cases (seizure types) observed between groups. For *p*-Value(*), the “Non-Deletion” group has ≤ 5 cases; therefore, the *p*-Value should be interpreted as indicative of a trend.

Sleep problems were described in 43 (69.4%) participants, 21 of whom were taking sleep-related medication (e.g., melatonin). Patients receiving sleep therapy had epilepsy, but no association between sleep problems and epilepsy was found. Participants reported difficulties with sleep onset and maintenance, with a median of 2 awakenings per night (IQR: 1.0–3.0). The prevalence of sleep disturbances did not significantly differ across genetic subtypes. Available data from 66 of the original 82 participants who completed the form on sleep are shown in Table 3.

The most frequently mentioned category of sleep problems was prolonged waking at night and co-sleeping. In two-thirds of patients (65.1%), participants described a medical condition that exacerbated sleep problems. In 53% of cases, participants rated sleep quality as moderate to severe.

Other heterogeneous symptoms (Table 1) were gastrointestinal problems [40 (64.5%), at least two of the main symptoms including drooling, reflux, or vomiting on feeding], skeletal/muscular disorders [30 (48.4%), including tight heel strands/toe walking and scoliosis], strabismus/other visual problems [42 (67.8%)], and pneumonia or other respiratory problems [27 (43.5%)].

## 4. Discussion

This paper intends to provide clear and useful insights into the clinical landscape of the clinical history and genetic characteristics of individuals with Angelman syndrome (AS) in Italy, utilizing data from the Italian Angelman Syndrome Registry (RISA). To our knowledge, this is the first study in Italy focusing on AS with the direct involvement of participants in the data collection process. However, this is not an attempt to study the epidemiology of AS in Italy and should not be considered representative of the true extent of the AS population in the country.

As there are limited outcome data for people living with a rare disease [39,40], patient/family involvement in reporting disease and treatment experiences is an important source of information [20,21,22,23,24,25,26,27,28,29,30,41].

Comparisons of the study sample with the global initiative, where Italian patients were poorly represented (Table S1), help in assessing the sample’s representativeness. However, the results should be interpreted cautiously, considering the limitations imposed by sample size imbalances and their potential implications for generalizability.

In terms of validating this initiative, there are still some challenges to overcome before it becomes a widely adopted research method.

The rationale for using a web-based approach was to maximize patients’ engagement across a large geographical area and overcome the problem of recruitment and retention of participants in traditional clinical studies. However, this approach appears to favor the recruitment of younger patients (similar to the Global Registry, with 54.9% of the participants aged <11 years), typically comprising individuals who have been recently diagnosed and who have fewer barriers to participation (i.e., individuals who are familiar with using an electronic data capture system) [34]. Further efforts are necessary to investigate why some participants have not completed their assessments and to determine the most effective and efficient methods for informing patients and families about registries and the significance of population-based research. Indeed, the direct involvement of patients may pose challenges in obtaining high-quality data and maintaining consistent data entry [34]. For instance, in RISA, the percentage of participants providing diagnostic information was 75.6%, in contrast to 30.0% in the Global Registry [34]. This variance stemmed from a 3-year evaluation period rather than a 1-year assessment. Nevertheless, implementing source data verification and providing feedback to participants could serve as strategies to mitigate potential issues with missing or misreported data.

Despite the underrepresentation of the Italian population in the Global Registry at the time of the report [34], the demographic and genetic data were aligned with the broader outcomes observed in the global initiative. The registry data confirmed the diagnostic delay of approximately 2 years and the distribution of genetic subtypes, with the distribution of genotypes showing that recent advances in genomics have improved the diagnosis of non-deletion subtypes, which is supported by existing studies [18,34,35].

Overall, in line with the literature on AS, the registry provided an overview of neurodevelopmental disorders (encompassing all reported cases) and described the clinical spectrum, with epilepsy (80–90% in the literature) and sleep disorders (20–80%) confirmed as the most common problems affecting the lives of AS patients and their parents [42,43,44,45]. The most common seizure types reported were generalized tonic–clonic seizures and atypical absence seizures, which is consistent with previous studies [36]. The data also corroborate a trend towards an association between 15q11–q13 deletion and the severity of manifestations, as well as shedding light on the frequency and severity of epilepsy [35,36]. Ascertaining the impact of epilepsy on the lives of AS patients is growing in significance, especially considering the valuable insights provided by participants on seizure-related observations [46]. Despite the fact that clinicians may overlook certain symptoms and the effects of seizures that caregivers might self-report [46], it is plausible that some seizures were underreported due to challenges in distinguishing seizure types, especially between focal motor seizures and generalized tonic–clonic seizures.

The data from the form on sleep disorders indicated shorter sleep duration and poorer sleep efficiency. The lack of a standardized definition of sleep disorders requires adept assessment methods and more detailed sample descriptions/analyses [31,46]. However, systematic collection of PROs [23,47] and providing feedback to participants may be recognized as opportunities for quality improvement in understanding health-related quality of life and the impact of symptoms on disease.

Future efforts should focus on adapting existing PROs or developing specific measures specifically tailored for the domain of Angelman syndrome to capture important outcomes for both patients and caregivers.

The RISA had both advantages and limitations compared to traditional data collection. The results should be interpreted in light of a modest sample of participants in the subgroups. However, the current number of participants matched the number of participants in previous studies [12,18,48]. We have chosen to conduct our analyses on participants for whom reliable data were collected, potentially affecting the alignment between our sample and the target population and thereby limiting the generalizability of our findings. Parent-reported data could bias the results. The reliability of self-reported data, the possibility of multiple reporting leading to duplicate records, and ethical considerations regarding possible intentional or unintentional misuse should be taken into account. In addition, some characteristics that contribute to the assessment of epilepsy severity, such as therapies, were not systematically recorded in the registry. These aspects should be taken into account when the results of studies conducted on the basis of patient self-reporting are transferred to the general population. Despite efforts to standardize variables related to epilepsy provided by families, it is important to acknowledge that these variables may not always align with the data reported by previous studies. For example, not all individuals with AS undergo imaging studies or have a clear therapeutic regimen, despite their availability for some patients. Other considerations are prospective for future initiatives; for example, data entry did not adhere to a fixed schedule for patient evaluations. Participants provided an overview of the patient in a single instance. Consequently, the data may vary depending on the patient’s age and the documentation provided, and did not enable tracking of long-term outcomes for individuals with AS in Italy.

## 5. Conclusions

In summary, the data comprehensively delineated the spectrum of clinical and genetic features of Angelman syndrome (AS) and confirmed the association between 15q11–q13 deletion and the severity of epilepsy manifestations within a sample of AS patients, emphasizing the frequency and severity of seizures. These findings align with the previous literature and highlight the importance of leveraging patients for data collection.

By promoting family involvement and implementing standardized data collection protocols, patient-driven registries provide the scientific community with valuable insights for future investigations and are a valuable model for improving research that can be applied to other rare diseases.

## Figures and Tables

**Table 1:** Sample description.

Demographic	*n* = 82
Age, median (IQR) ^1^	8.0 (3.0–17.7)
Male gender, *n* (%)	40 (48.8)
Origin (Caucasian) ^2^, *n* (%)	81 (98.8)
Birth-related complications [Y(%)/N/NA] ^3^	19 (23.2)/50/13
Pregnancy-related complications [Y(%)/N/NA]	18 (21.9)/51/13
**Motor and Speck Skills (age > 2 y)** ^4^	***n* = 69**
Age at unsupported sitting (months), median (IQR)	12.0 (9.0–16.0)
Age of standing and initial steps (months), median (IQR)	32.0 (24.0–39.7)
Expressive language, *n* (%)	57 (83.0)
**Associated Clinical Manifestations**	***n* = 62**
Age of diagnosis (months), median (IQR)	23.0 (13.0–36.0)
Hospitalization for comorbidities (>2 events), *n* (%)	34 (55.0)
Deletion, *n* (%)	36 (58.1)
Epilepsy	51 (82.2)
Age of first seizure (months), median (IQR)	30.0 (24.7–36.0)
Response to anti-epileptic drugs (AEDs), [Y(%)/N/UNK]	39 (90.7)/4/1
Sleep problems, *n* (%)	43 (69.4)
Strabismus/other vision problems, *n* (%)	42 (67.8)
Gastrointestinal problems, *n* (%) ^5^	40 (64.5)
Drooling	54 (87.0)
GER	41 (66.1)
Vomiting with feeds	28 (45.2)
Constipation	47 (75.8)
Skeletal/muscle disorders, *n* (%) ^6^	30 (48.4)
Respiratory problems, *n* (%)	27 (43.5)
Pneumonia	11 (17.7)

Data are presented as a median with interquartile range (IQR), a number with percentage (%), or the number (%) of positive (Y)/negative (N)/missing (NA) or Unknown(UNK). Sources of information: demographic data refer to participants who completed the registration process (*n* = 82); ^1^ Age at the time of registration/end of data entry; ^2^ Ethnicity of parents was taken into account. ^3^ Hospitalizations in neonatal pathology during the first 12 months of life. ^4^ Assessment in patients with age > 2 years at the time of registration. Genotype and associated medical manifestations are from 62 patients who also provided a complete set of information. ^5^ Gastrointestinal problems (at least two major symptoms, including drooling, reflux, or vomiting with feeds). GER (Gastroesophageal reflux). ^6^ Skeletal/muscle disorders (including tight heel cords/toe walking and scoliosis).

**Table 3:** Parent-reported sleep problems.

Sleep Problem	*n* = 66
**Insomnia, *n* (%)**	
Daytime sleepiness (≥2)	9 (3.6)
Night sleep latency (≥30 min)	33 (50.0)
Night wakings (≥2)	32 (48.5)
Co-sleeping	46 (69.7)
**Quality of sleep, *n* (%)** ^1^	
No problem	7 (10.6)
Mild	24 (36.4)
Moderate	21 (31.8)
Severe	14 (21.2)
**Impact on quality, *n* (%)**	
Illness	43 (65.1)
Fatigue	9 (13.6)
Change in daytime routine	25 (37.9)

The form refers to the last years of patients (at the time of registration/completion of data). ^1^ Ten-grade Visual Analogue Scale (VAS).

## Data Availability

All data generated or analyzed as part of this study are included in this published article. The data sets generated and analyzed are not publicly available, as informed consent only allows the publication of aggregated data.

## Supplementary Materials

The following supporting information can be downloaded at: https://www.mdpi.com/article/10.3390/jcm13123520/s1; Supplementary S1: Figure S1: Flowchart of the registration process; Table S1: Demographic and clinical characteristics of the participants included in RISA vs. the Global Registry.
