# Supplementary material for: Exploring the Clinical and Genetic Landscape of Angelman Syndrome: Patient-Reported Insights from an Italian Registry
Source: J Clin Med. 2024 Jun 16;13(12):3520. doi: 10.3390/jcm13123520 (PMC11204522; doi:10.3390/jcm13123520)
Supplement: Supplementary file 1 [file jcm-13-03520-s001.zip › jcm-3018651-supplementary.pdf]

Journal of
Clinical Medicine

MDPI

Supplementary S1

Supplementary Methods

Development of the registry

Representatives of patient organizations and families have supported the implementation of the registry and have been involved since the beginning of the registry's activities. Participants were reached through patient organizations and an online website. Collaboration with AS associations played an important role in the recruitment of patients. They helped to disseminate information about the registry and encouraged their members to participate.

By its objectives, the registry can be classified as a public registry investigating the natural history and geographical distribution of AS [29]. The registry fulfils the following classification criteria: 1) disease-based; 2) user-driven (only parents/guardians can provide information); 3) electronic-based; 4) based on geographic coverage [30]. The study protocol underwent an internal validation process which involved discussions with experts in rare diseases and AS. Subsequently, a preliminary test was then conducted with association participants to identify any issues or ambiguities. Based on the feedback received, necessary changes or adjustments were made to optimize the protocol for consistent and accurate data collection.

Structure of the Registry

Signing the informed consent, the participating user (parent/guardian) agreed to receive information about the purpose of the register, the type of information collected, the use of the data and data access governance. Security measures for handling personal and sensitive data were well established and guaranteed by a high level of protection (see below).

Platform and workflow

The project page is available online (www.registroitalianoangelman.it). The registry was created using Electronic Data Capture technology. Data were entered into electronic forms directly by the participants through their mobile devices or computers via a web/mobile-based interface. Essentially, the following measures were taken to manage sensitive data: 1) security and multi-level access for users and team members; 2) storage of participants' personal data in a separate area with automatically generated pseudo-anonymisation codes; 3) data storage on a dedicated server. Each team member had access to the system with personal credentials and according to their role via a traceable procedure. Once consent was granted, participants were given full access to a secure, personalized homepage (Fig. S1).

Work team

The work team was responsible for the registry content and the coordination of all operational activities. The team had different expertise specific to each aspect of the registry, including a Medical Project Manager (or Registry Curator – responsible for reports, communication, strategy, relations with collaborators); a Neurologist (responsible for data supervision); a Data Manager (responsible for data management and monitoring); a Biostatistician (responsible for database development, data analysis and interpretation); Information Technology personnel (with tasks such as technical development of the registry platform and bug resolutions). Experts in RDs and AS were also involved at various levels, including consultation in data review and contributing to intellectual content during manuscript preparation.

Data collection

Standardized forms were employed, alongside the centralization of source documents (validated copies of the original documents). The electronic forms consisted of a "semi-structured data entry" format with guided items (such as checkboxes or radio buttons) designed to capture relevant information regarding participants' demographic characteristics, medical history, diagnosis, genetic results, behavior, development, sleep patterns, and other pertinent aspects related

to AS. Additionally, participants could fill in limited text fields. Validated questionnaires were not utilized because there were no formally validated questionnaires specifically for AS in the literature at the time RISA was launched. However, common items identified in the scientific literature were included, as there are no formal common data elements (CDEs) specifically for AS.

Quality Control and Validation

Of the 93 respondents, 82 met the inclusion criteria and were able to enter data into the registry. The remaining 11 respondents were excluded because the diagnosis of AS was not confirmed or informed consent was not obtained. Participants who had filled on data and locked user access were considered completed (n=62). A major challenge of the registry was ensuring the reliability and quality of the data, especially given the user-driven information. This challenge was addressed by redirecting to a landing page with detailed information about the objectives of the study and instructions for completing the forms, as well as a centralized review of source documents ((validated copies of the original documents) by team experts and data feedback to participating users (e-queries resolutions). Participants did not update their information in real-time. Instead, they submitted a historical record of data at enrollment, which was then stored in the registry. This approach allowed families to report symptoms, treatments, and other relevant information based on the patient's condition at the time of data collection, rather than adhering to fixed schedules of clinic visits.

To enhance the accuracy of the self-reported data and mitigate potential biases, several measures were implemented.

Automatic data entry verification: when participants entered the medical records, the system prompted them to provide supporting documentation, such as genetic test results or physician's notes. Automatic data quality checks were included to flag inconsistent or incomplete entries.

Standardized forms: Participants completed standardized forms designed to cover key areas.

Data review: study team reviewed the submitted records to verify the accuracy of the medical information. This process involved cross-checking medical records or request for additional information if necessary.

Ongoing monitoring: continuous monitoring and periodic audits of the entered data were performed by the study team. This process included regular checks with participants to review and confirm the accuracy of their medical records.

Figure S1. Flowchart of the registration process

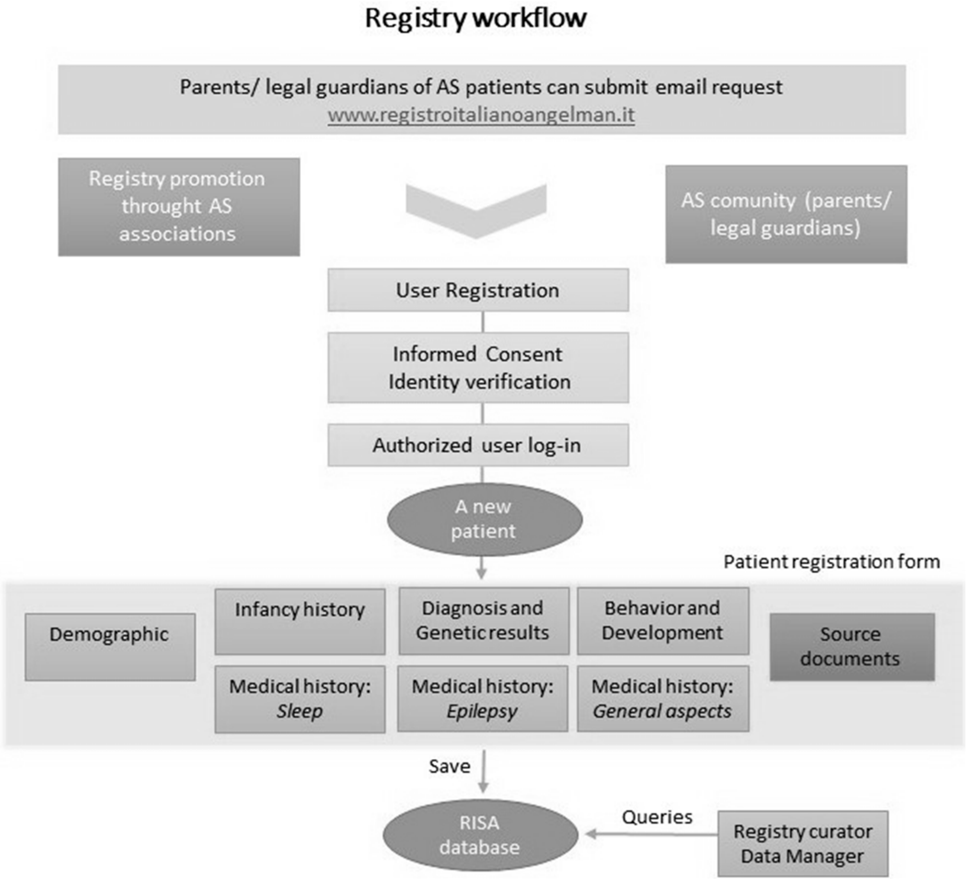

Modular structure and data collected. Data are encrypted and stored. Demographic data are stored separately.

Table S1. Demographic and clinical characteristics of the participants included in RISA vs the Global Registry

		RISA	Global Registry	p-value
Demographic		n=82	n=467	
Age group (yrs), n (%)	0-2	13 (15.9)	65 (14.0)	0.643
	3-5	19 (23.2)	107 (23.0)	0.959
	6-10	13 (15.9)	98 (21.0)	0.286
	11-15	12 (14.6)	79 (17.0)	0.608
	16-20	10 (12.2)	40 (8.6)	0.292
	21-30	10 (12.2)	47 (10.0)	0.560
	>30	5 (6.1)	27 (5.7)	0.803
Male gender, n (%)		40 (48.8)	234 (50.0)	0.825
Genotype		n=62	n=140	
Deletion		36 (58.1)	106 (76.0)	0.018
Uniparental paternal disomy		10 (16.1)	10 (7.1)	0.090
Mutation		11 (17.7)	13 (9.3)	0.139
Imprinting defect		2 (3.2)	5 (3.8)	-
Abnormal DNA methylation/ undefined cases		3 (4.8)	4 (3.0)	-

Chi-Square Test (or Fisher-Test when appropriate) is used.
